# High-Intensity Interval Training Is Associated With Alterations in Blood Biomarkers Related to Brain Injury

**DOI:** 10.3389/fphys.2018.01367

**Published:** 2018-09-28

**Authors:** Alex P. Di Battista, Katherine A. Moes, Maria Y. Shiu, Michael G. Hutchison, Nathan Churchill, Scott G. Thomas, Shawn G. Rhind

**Affiliations:** ^1^Defence Research and Development Canada, Toronto Research Centre, Toronto, ON, Canada; ^2^Faculty of Kinesiology & Physical Education, University of Toronto, Toronto, ON, Canada; ^3^Neuroscience Program, Keenan Research Centre for Biomedical Science of St. Michael’s Hospital, Toronto, ON, Canada

**Keywords:** tau, PRDX-6, S100B, sport concussion, military medicine, MTBI

## Abstract

**Purpose:** Blood biomarkers are a useful tool to study concussion. However, their interpretation is complicated by a number of potential biological confounds, including exercise. This is particularly relevant in military and athletic settings where injury commonly occurs during physical exertion. The impact of high-intensity interval training (HIIT) on putative brain injury biomarkers remains under-examined. The purpose of this study was to observe the effects of HIIT on a panel of blood biomarkers associated with brain injury.

**Methods:** Eleven healthy, recreationally active males (median age = 29.0, interquartile range = 26.0–31.5) performed HIIT on a bicycle ergometer (8-12 × 60-s intervals at 100% of peak power output, interspersed by 75-s recovery at 50 W) three times/week for 2 weeks. Peripheral blood samples were collected before and immediately after HIIT during the first and last training sessions. Plasma concentrations of s100 calcium-binding protein beta (S100B), glial fibrillary acidic protein (GFAP), neuron-specific enolase (NSE), brain-derived neurotrophic factor (BDNF), neurogranin (NRGN), peroxiredoxin (PRDX)-6, creatine kinase-BB isoenzyme (CKBB), visinin-like protein (VILIP)-1, von Willebrand factor (vWF), monocyte chemoattractant protein (MCP)-1, matrix metalloproteinase (MMP)-9, and total tau (T-tau) were quantitated by high-sensitivity MULTI-SPOT^®^ immunoassay, on the MesoScale Diagnostics electrochemiluminescence detection platform. Differences in biomarker concentrations in response to HIIT were evaluated by partial least squares discriminant analysis (PLSDA) within a repeated-measures bootstrapped framework.

**Results:** Ten of 12 biomarkers were increased pre-to-post HIIT; VILIP-1 remained unchanged, and GFAP was not statistically evaluated due to insufficient detectability. After 2 weeks of HIIT, T-tau was no longer significantly elevated pre-to-post HIIT, and significant attenuation was noted in the acute responses of NRGN, PRDX-6, MMP-9, and vWF. In addition, compared to session 1, session 6 pre-exercise concentrations of NSE and VILIP-1 were significantly lower and higher, respectively.

**Conclusion:** Blood biomarkers commonly associated with brain injury are significantly elevated in response to a single bout of HIIT. After a 2-week, six-session training protocol, this response was attenuated for some, but not all markers. While biomarkers continue to provide promise to concussion research, future studies are necessary to disentangle the common biological sequelae to both exercise and brain injury.

## Introduction

Blood biomarkers are a promising tool to investigate concussion and other mild forms of brain trauma. They hold potential clinical utility in diagnosis, prognosis, and therapeutic decision-making, and can be used to elucidate secondary injury pathophysiology in human studies (Wang et al., 2018). However, as opposed to severe traumatic brain injury (TBI) where peripheral blood biomarker concentrations can acutely fluctuate by several orders of magnitude (Di Battista et al., 2016a), the relatively modest changes observed in concussion are often more difficult to characterize. Moreover, concussions occur frequently in the athletic and military settings where injury commonly accompanies physical exertion (Garber et al., 2014, 2016). This presents both methodological and analytical challenges, as exercise itself elicits profound physiological and biochemical alterations, and many of these perturbations are similar to those seen in concussion and/or mild head trauma (Reihmane et al., 2012; Cook et al., 2013; Koh and Lee, 2014; Stocchero et al., 2014; Bessa et al., 2016; Kaspar et al., 2016; He et al., 2017). Hence, the continued use of blood biomarkers for concussion in physically active populations necessitates a better understanding of how they may be affected by both acute and chronic exercise (Broglio et al., 2017).

Broadly, biomarkers used in brain injury research can be categorized into those that reflect injury to neural tissue (i.e., neuronal, axonal, and glial cells), which are labelled as “brain-specific,” and those that reflect important secondary injury processes that are characteristic of, but not limited to, concussion. The latter typically includes inflammation, oxidative stress, and vascular endothelial injury. Indeed, there is supportive evidence that biomarkers related to each of the aforementioned processes can be acutely augmented with exercise (Kawata et al., 2016). For example, the most widely studied brain injury biomarker, S100B, can be elevated in the blood after both exercise and sport participation, irrespective of brain trauma (Dietrich et al., 2003; Hasselblatt et al., 2004; Stocchero et al., 2014). Furthermore, while inflammation and oxidative stress are both crucial manifestations of secondary injury after concussion, they are also prominent features of the acute physiological response to physical exertion (Inkabi et al., 2017). Indeed, circulating levels of inflammatory cytokines such as tumor necrosis factor (TNF)-α, interleukin (IL)-6, and monocyte chemoattractant protein (MCP)-1, which are also well-studied in TBI, increase after aerobic and anaerobic exercise (Ostrowski et al., 1999; Reihmane et al., 2013; Kim et al., 2015; Bessa et al., 2016). In addition, post-exercise perturbations in blood indices of oxidative stress have been identified, including those related to lipid peroxidation and reactive oxygen species production (Brinkmann et al., 2012; Turner et al., 2013; Wadley et al., 2015; Roh et al., 2017). Finally, increased blood concentrations of matrix metalloproteinase (MMP)-9, an indicator of extracellular matrix degradation and vascular remodelling, have been observed after both aerobic and maximal exercise (Reihmane et al., 2012, 2013).

The use of biomarkers to study concussion in habitually active or highly trained populations also requires cognizance of the chronic effects of physical activity on circulating biomarker levels. Evidence suggests that prolonged exercise (weeks-to-months) is associated with lower resting concentrations of biomarkers related to neurodegeneration (Liang et al., 2010) inflammation (Koh and Park, 2017), oxidative stress (Bloomer and Fisher-Wellman, 2008; Cook et al., 2013), and vascular endothelial function (Cook et al., 2013). However, the interpretation and utility of these findings is limited by our incomplete understanding of the mechanisms by which either brain injury or exercise alters blood biomarker concentrations. Unsurprisingly, it appears that there are shared mechanisms underlying biomarker changes in both conditions. For example, evidence from animal model studies suggests that both physical activity and TBI alter glympathic clearance rates (Plog et al., 2015; He et al., 2017), and in the case of TBI, glymphatic clearance may be the primary mechanism through which molecular indices of brain damage appear in the peripheral circulation (Plog et al., 2015). Furthermore, both exercise (Sharma et al., 1991; Watson et al., 2005; Roh et al., 2017) and brain injury (Chodobski et al., 2011) may disrupt the blood-brain barrier (BBB), possibly leading to the leakage of molecules from the brain parenchyma into the periphery (Nierwińska et al., 2008). Lastly, and with particular relevance to biomarkers of inflammation, both exercise (Fu and Levine, 2013) and concussion (Blake et al., 2016; Esterov and Greenwald, 2017) alter autonomic nervous system function. The resultant shifts in sympathetic/parasympathetic balance can directly influence immunoinflammatory responses in various ways, including *via* interactions between stress hormones and circulating immune cells, and neural activation of the acute phase response by the liver (Elenkov et al., 2000; Catania et al., 2009). In both instances, peripheral inflammatory cytokine and chemokine concentrations can be altered.

High-intensity interval training is an attractive exercise paradigm to study the effects of physical exertion on blood biomarkers in athletic and military populations (Gibala et al., 2015). The inherent variation in intensity may accurately portray sport participation and military operations, yet unlike these situations, is administered in structured (intensity and duration) and easily quantifiable sessions (Kyrolainen et al., 2017). Indeed, deriving samples from military personnel during or after military operations can be difficult and often impractical, and the quantitation of biomarkers after sport participation is also challenging due to the inherent subject-to-subject variance in the intensity and degree of physical stress. Furthermore, when investigating neural injury biomarkers, their quantitation after sport participation may be confounded by subconcussive impacts (Broglio et al., 2017) and/or the potential biological perturbations associated with musculoskeletal injury.

There is limited research examining the effects of HIIT on circulating biomarkers relative to other forms of exercise (Leggate et al., 2010; Rakobowchuk et al., 2012; Wahl et al., 2014; Zwetsloot et al., 2014; Dorneles et al., 2016; Kaspar et al., 2016). In particular, there is a paucity of research on the effects of HIIT on markers related to brain injury, with only a single published report of elevations in serum brain-derived neurotrophic factor (BDNF) acutely after exercise (Saucedo Marquez et al., 2015). Therefore, the purpose of this controlled laboratory study was to characterize a panel of blood biomarkers associated with brain injury in response to an acute bout of HIIT, before and after a training period.

## Materials and Methods

### Participants

Eleven physically active adult males [mean ± SD age: 28.8 ± 5.3 years; body mass: 82.4 ± 7.6 kg; height: 179.0 ± 8.6 cm; body fat: 17.2 ± 5.2%; relative peak oxygen uptake (VO_2peak_): 43.3 ± 4.4 ml kg^-1^ min^-1^] volunteered to participate in the study. Healthy, male, recreationally active (i.e., engage in purposeful exercise at least once per week, but do not cycle regularly) Canadian Armed Forces (CAF) members between the ages of 20 and 40 years were included in the study. Exclusion criteria consisted of the presence of acute and chronic diseases, the use of prescription or over-the-counter medications, a history of concussion, blood donation within the last 3 months, or smoking. Before commencing testing, volunteers were given a full explanation of the study procedures, including all possible risks and discomforts associated with the study, and provided their written informed consent; the research protocol was approved by the Defence Research and Development Canada Human Research Ethics Committee and University of Toronto Research Ethics Board in accordance with the Declaration of Helsinki.

### Experimental Design and Procedures

All participants were asked to visit the DRDC TRC laboratory on a total of nine occasions. All testing was carried out under controlled lab conditions (temperature: 24 ± 1°C; relative humidity: 30 ± 2%). At the *first session*, a pre-participation medical screening was performed by a licenced physician, a Physical Activity Screening Questionnaire of habitual exercise habits was completed, anthropometric (height and weight) and body composition (fat mass and fat free mass) measurements were made, and participants were familiarized with the phlebotomy equipment and high-intensity cycling protocol to be performed in the following sessions. All subjects were asked to refrain from strenuous physical activity for 48 h prior to the beginning of the study, and not to exercise outside of the study protocol for the duration of the study. For the *second session*, participants came to the lab 1 day later for a baseline maximal graded VO_2peak_ exercise test on a cycle ergometer. Within 30 days of the completion of VO_2peak_ testing, participants returned to the lab (*session 3*) for the first HIIT-(1) exercise session. At this time, resting pre-exercise and post-exercise peripheral blood samples were taken for the measurement of selected plasma biomarkers. Over the next 2 weeks, participants completed an additional five HIIT bouts (*sessions 4*-*8*) with pre- and post-exercise blood sampling only during *session 8* (HIIT-6). During *session 9*, participants completed a second post-training maximal graded VO_2peak_ test, identical to *Session 2*.

### Anthropometric and Body Composition Measurements

Height (cm) was recorded using a stadiometer in standing position close to the wall without shoes while the shoulders of participants were in normal position. Body mass (kg) was measured without shoes and minimal clothing using a BOD POD^TM^ digital electronic scale. Body fat measurements were determined non-invasively using the BOD POD^TM^ Air Displacement Plethysmograph (COSMED USA, Inc., Concord, CA, United States), according to the manufacturer’s guidelines.

### Maximal Graded Exercise Test

A continuous incremental exercise test to exhaustion was performed on a computer-controlled electronically braked cycle ergometer (Velotron Dynafit Pro, RacerMate Inc., Seattle, WA, United States), to determine each participant’s VO_2peak_ and peak work rate (*W*_peak_) in Watts (W). Participants were fitted with a heart rate monitor (Polar WearLink^®^+, Polar Electro Canada, Lachine, QC, United States) to record resting heart rate prior to testing and continuously during exercise. After a 2-min warm-up at a resistance of 50 W, the workload was increased by 25 W every 60 s until the participant reached volitional exhaustion. Oxygen uptake was obtained through ventilation and respiratory gas exchange analysis sampled continuously using an automated metabolic analyzer (MOXUS Modular Metabolic System, AEI Technologies, Inc., Pittsburgh, PA, United States). The exercise was considered maximal when a plateau in oxygen uptake, respiratory exchange ratio, or age-adjusted maximal heart rate was achieved. VO_2peak_ was determined as the maximum oxygen uptake over a 15-s averaging analysis and *W*_peak_ corresponded to the highest resistance maintained over a 60-s period during the test. Maximal heart rate was measured at exhaustion. Ratings of perceived exertion (RPE; Borg category-ratio 10 scale) were recorded every 2 min. Participants received continuous verbal encouragement during the test. A second post-training VO_2peak_ test using an identical protocol was performed within a minimum of 24 h, but no more than 48 h after completion of the last HIIT session.

### High-Intensity Interval Training

All training was performed on the same cycle ergometer used for the incremental exercise test. A minimum of 48 h and not more than 30 days following the initial VO_2peak_ test, participants commenced a 2-week HIIT regimen. Sessions took place on the Monday, Wednesday, and Friday of two consecutive weeks. Each session was performed between 0800 and 1000 hours, after an overnight fast (∼10 h). The HIIT protocol consisted of a 3-min warm-up at 50 W followed by eight repeated 60-s intervals of cycling during HIIT-1 and HIIT-2, 10 intervals during HIIT-3 and HIIT-4, and 12 intervals during HIIT-5 and HIIT-6, at *W*_peak_, interspersed with 75-s active recovery periods at 50 W. Participants completed the protocol with a 3-min cool down at 50 W and maintained the 60 rpm cadence throughout the sessions.

### Blood Sampling and Biomarker Analysis

Venous blood samples were obtained just before exercise from resting subjects and immediately post-exercise on HIIT-1 and HIIT-6, using standard phlebotomy techniques. Specimens were drawn directly into 3 mL K_2_EDTA vacutainers (BD Vacutainer^®^, Franklin Lakes, NJ, United States). Blood samples were immediately centrifuged at 1600 ×*g* for 15 min at 4°C after collection and plasma supernatant was then aliquoted and frozen at -70°C until analysis. All samples were processed in the same manner.

Plasma biomarkers levels were evaluated by Meso Scale Diagnostics, LLC (MSD) using prototype 96-well MULTI-ARRAY^®^ immunoassay plates developed in part through work supported by US Army Medical Research and Material Command (Contract W81XWH-13-C-0196). Plasma concentrations (pg/ml) of glial fibrillary acidic protein (GFAP), s100B, neuron-specific enolase (NSE), total-tau (T-tau), neurogranin (NRGN), creatine kinase-BB isoenzyme (CKBB), visinin-like protein (VILIP)-1, von Willebrand factor (vWF), BDNF, peroxiredoxin (PRDX)-6, MMP-9, and MCP-1 were quantified in duplicate on a SECTOR^®^ Imager 6000 reader using electrochemiluminescence detection (Debad et al., 2004) in an array-based multiplex format, as previously reported (Di Battista et al., 2016b, 2018; Kilianski et al., 2017). Hematological characteristics were measured using the COULTER AcT diff2^TM^ (Beckman Coulter Inc., Brea, CA, United States), and biomarker levels were corrected for plasma volume shift using the equations developed by Dill and Costill (1974).

### Statistics

Participant performance measures taken before and after six sessions of HIIT training were evaluated *via* non-parametric Wilcoxon signed-rank tests, corrected for multiple comparisons at a false discovery rate (FDR) of 0.05. A partial least squares discriminant analysis (PLSDA) within a repeated-measures resampling framework was used to evaluate biomarker concentrations in relation to HIIT. Briefly, PLSDA is a supervised, multivariate technique used to identify linear combinations of predictor variables (e.g., blood biomarkers) that have maximal covariance with a binary outcome (e.g., pre-/post-HIIT status). The PLSDA analysis produces loadings (i.e., saliences) on predictor variables reflecting their contribution to this multivariate relationship. We identified significant saliences by bootstrap resampling on subjects (5000 iterations) and generated empirical *p*-values based on the fraction of the bootstrap distribution that overlaps zero. Significant *p*-values were reported after adjusting for multiple comparisons at a FDR of 0.05. The effect size for biomarker saliences was also reported as the bootstrap ratio of each variable (mean/bootstrapped standard error). In the current study, three PLSDA tests were administered: (1) to investigate the acute effect of HIIT on biomarker concentrations, pre vs. post HIIT biomarkers were compared at both session 1 and session 6; (2) to test for changes in the acute biomarker response to HIIT before and after six sessions of training, difference scores were calculated for each biomarker by subtracting the pre-HIIT value from the post-HIIT value, and then comparing the difference scores of session 1 vs. session 6; and (3) to test for changes in baseline biomarker concentrations prior to and after 6 sessions of HIIT, pre-HIIT values were compared at session 1 vs. session 6. Prior to PLSDA analysis, missing biomarker values were imputed using the variable mean, and data were rank-transformed to ensure robustness against non-normality; individual biomarkers were excluded if >20% of the values were missing, or if there were >5% missing values across all biomarkers. Percent detectability information for each biomarker can be seen in **Supplementary Table S1**, and assay performance for each biomarker can be seen in **Supplementary Table S2**.

All statistical analyses were conducted using R (RStudio, version 1.1.383, Boston, United States). Graphs were prepared using GraphPad Prism (version 7.0d, GraphPad Inc., La Jolla, CA, United States).

## Results

### Description of Participants

Participant pre-training characteristics and performance measures are summarized in **Table 1**. Briefly, six sessions of HIIT training was associated with significant increases in peak oxygen uptake (VO_2peak_ = 42.4 vs. 44.3 ml kg^-1^ min^-1^, pre vs. post training, *p* = 0.045) and peak power production (*W*_peak_ = 325.0 vs. 350.0 W, pre vs. post training, *p* = 0.018).

**Table 1:** Pre-training subject characteristics.

Variable	Median and IQR (*n* = 11)
Age (years)	29.0 (26.0–31.5)
% Male	100.0
Height (cm)	184.0 (178.6–185.4)
Weight (kg)	80.2 (77.9–87.7)
Body fat (%)	17.9 (14.4–19.9)
VO_2peak_ (ml kg^-1^ min^-1^)	42.4 (39.6–46.4)
Peak power (W)	325.0 (287.5–333.7)


*IQR, interquartile range; VO_2peak_ (ml kg^-*1*^ min^-*1*^), peak oxygen uptake.*

### Blood Biomarkers

**FIGURE 1:**
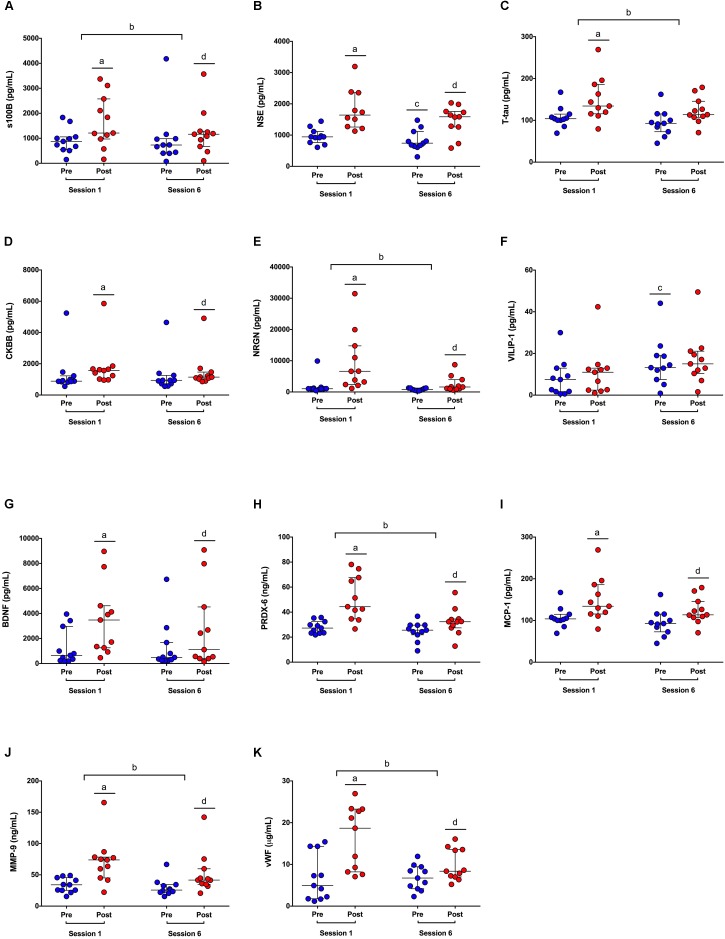
Blood biomarker concentrations in response to HIIT. **(A–K)** S100 calcium binding protein beta (S100B); neuron-specific enolase (NSE); total tau (T-tau); creatine kinase-BB isoenzyme (CKBB); neurogranin (NRGN); visinin-like protein (VILIP); brain derived neurotrophic factor (BDNF); peroxiredoxin (PRDX); monocyte chemoattractant protein (MCP); matrix metalloproteinase (MMP); von Willebran factor (vWF). Dots represent blood biomarker concentrations pre (blue dots) and post (red dots) exercise, at sessions 1 and 6. Significant differences in biomarker concentrations post-exercise vs. pre-exercise at session 1^a^, pre-post difference scores at session 6 vs. session 1^b^, pre-exercise at session 6 vs. session 1^c^, and post-exercise vs. pre-exercise at session 6^d^, by PLSDA analysis, corrected at FDR = 0.05.

Biomarker concentrations at each time-point can be found in **Table 2**, and bootstrap ratios reflecting biomarker effect sizes for each PLSDA analysis can be found in **Supplementary Table S3**. GFAP was excluded from statistical analysis as it was not detectable in >80% of sample (**Supplementary Table S1**). Blood concentrations for all biomarkers at each time-point can be seen in **Figure 1**. Participants’ first HIIT training session was associated with significant increases seen immediately after exercise in all biomarkers except VILIP-1. The greatest effects were observed for NSE (bootstrap ratio = 11.3; 943.0 vs. 1641.6 pg/mL pre vs. post, respectively, **Figure 1B**), NRGN (bootstrap ratio = 9.2; 1031.2 vs. 6627.5 pg/mL pre vs. post, respectively, **Figure 1E**), and PRDX-6 (bootstrap ratio = 8.8; 27.2 vs. 44.3 ng/mL pre vs. post, respectively, **Figure 1H**). Participants’ sixth training session was associated with significant increases immediately after exercise in all biomarkers except VILIP-1 and T-tau. The largest effects were seen in NRGN (bootstrap ratio = 5.8; 786.5 vs. 1601.8 pg/mL pre vs. post, respectively, **Figure 1E**), vWF (bootstrap ratio = 5.4; 6.7 vs. 8.4 μg/mL pre vs. post, respectively, **Figure 1K**), and MMP-9 (bootstrap ratio = 4.9; 25.6 vs. 41.6 ng/mL pre vs. post, respectively, **Figure 1J**). In addition, compared to session 1, the acute increases observed in numerous biomarkers were attenuated at session 6. The most significant attenuations were seen in PRDX-6 (bootstrap ratio = -6.1; median Δ = 16.1 vs. 4.5 ng/mL in HIIT session 1 vs. session 6, respectively, **Figure 1H**), NRGN (bootstrap ratio = -5.2; median Δ = 4866.2 vs. 493.2 pg/mL in HIIT session 1 vs. session 6, respectively, **Figure 1E**), and vWF (bootstrap ratio = -4.4; median Δ = 8.4 vs. 4.0 μg/mL in HIIT session 1 vs. session 6, respectively, **Figure 1K**). Changes in biomarker concentrations pre-post HIIT at session 1 and session 6 can be seen in **Supplementary Table S4**. Finally, six sessions of HIIT training was associated with a significant increase in pre-exercise blood concentrations of VILIP-1 (bootstrap ratio = 2.7; 5.0 vs. 13.8 pg/mL pre-HIIT session 1 vs. session 6, respectively, **Figure 1F**), and a significant decrease in pre-exercise concentrations of NSE (bootstrap ratio = -3.1; 943.0 vs. 742.6 pg/mL pre-HIIT session 1 vs. session 6, respectively, **Figure 1B**).

**Table 2:** Biomarker values pre- and post-acute HIIT exercise, at sessions 1 and 6.

	HIIT Session 1 (n = 11)		HIIT Session 6 (n = 11)	
Biomarkers	Pre	Post	Pre	Post
s100B	875.8 (610.5–1027.2)	1209.3 (1051.3–2341.1)	731.7 (421.8–974.2)	1162.2 (807.4–1266.9)
NSE	943.0 (840.7–1044.0)	1641.6 (1332.0–2213.3)	742.6 (657.5–960.1)	1586.0 (1272.4–1742.0)
T-tau	12.5 (11.2–14.7)	21.4 (15.5–28.3)	14 (12.5–16.6)	16.6 (13.2–20.8)
CKBB	882.3 (853.6–978.6)	1566.0 (1130.6–1659.7)	939.5 (723.7–1099.6)	1146.9 (1052.1–1345.7)
NRGN	1031.2 (944.0–1158.9)	6627.5 (2804.8–12,888.6)	786.5 (540.2–1202.2)	1601.8 (1037.1–2921.0)
VILIP-1	5.0 (1.7–8.9)	8.85 (2.4–12.3)	13.8 (8.6–18.9)	16.0 (10.8–20.7)
BDNF	650.5 (294.2–1982.2)	3480.7 (1307.1–4371.7)	489.1 (277.8–1240.4)	1110.1 (471.4–3605.6)
PRDX-6 (ng/mL)	27.2 (23.4–31.1)	44.3 (38.1–66.2)	25.6 (23.1–29.5)	32.3 (28.5–34.3)
MCP-1	103.9 (100.2–111.2)	134.2 (117.6–174.5)	92.8 (78.4–107.7)	113.5 (108.5–136.0)
MMP-9 (ng/mL)	34.0 (25.4–43.4)	73.8 (52.3–77.8)	25.6 (22.7–33.5)	41.6 (35.4–52.1)
vWF (μg/mL)	4.9 (2.0–10.9)	18.7 (8.7–23.0)	6.7 (4.5–8.9)	8.4 (7.1–13.5)


*Biomarker concentrations reported in pg/mL unless otherwise stated, with values representing the median and interquartile range. s100B, s100 calcium-binding protein beta; NSE, neuron-specific enolase; PRDX, peroxiredoxin; GFAP, glial fibrillary acidic protein; BDNF, brain-derived neurotrophic factor; MMP, matrix metalloproteinase; MCP, monocyte chemoattractant protein; T-tau, total tau; CKBB, creatine kinase-BB isoenzyme; NRGN, neurogranin; VILIP, visinin-like protein; vWF, von Willebran factor.*

## Discussion

The main finding of the current study is that an acute bout of HIIT significantly increases blood concentrations of several putative brain injury-related biomarkers. Furthermore, we observed that a modest, 2-week training period can alter both resting and exercise-induced biomarker concentrations.

Ten of 12 biomarkers evaluated in this study were significantly elevated immediately after a single session of HIIT in physically active male participants; only VILIP-1 concentrations remained unchanged. A number of the neural biomarkers assessed in the current study have not been previously measured in the blood after exercise (CKBB, NRGN, and PRDX-6), and even fewer have been evaluated in response to in HIIT. Our results are in agreement with previous studies that, independent of brain trauma, have observed increases in s100B following aerobic exercise (Stocchero et al., 2014), and increases in s100B and/or NSE after a sporting event (Dietrich et al., 2003; Hasselblatt et al., 2004; Kiechle et al., 2014; Shahim et al., 2015). However, that we found a significant elevation in T-tau, but not a significant elevation in VILIP-1 after HIIT is in contrast with the findings of Shahim et al. (2015), who showed that a “friendly” game of hockey resulted in significant increases in VILIP-1 concentrations in the blood, but no change in T-tau. It is possible that the time of acquisition [1-h post exercise (Shahim et al., 2015) vs. immediate (current study)], and training stimulus [friendly game of hockey (Shahim et al., 2015) vs. HIIT (current study)] may account for these discrepancies. However, while we observed elevated concentrations of T-tau after the first HIIT session, at the sixth session, in agreement with Shahim and colleagues, we found no significant difference in pre vs. post exercise T-tau concentrations. These findings together support the potential effect of training on the attenuation of the T-tau response to exercise, as Shahim et al. (2015) employed trained professional hockey players, and we observed an attenuation after a training period in recreationally active subjects. Taken together, our results support the growing body of literature which suggests that biomarkers predominantly expressed within the central nervous system can be found in increased concentrations in the blood after exercise.

In the current study, the inflammatory chemokine MCP-1, the oxidative marker PRDX-6, and vascular endothelial indices MMP-9 and vWF were significantly elevated immediately after exercise. This is in agreement with previous HIIT studies that have found acute elevations in indices of each of the aforementioned categories: inflammation, endothelial vascular activation, and oxidative stress (Leggate et al., 2010; Wahl et al., 2014; Zwetsloot et al., 2014; Wadley et al., 2015; Souza-Silva et al., 2016). While PRDX-6 has not previously been evaluated in HIIT, increased protein expression of thioredoxin has been observed in peripheral blood mononuclear cells after low intensity interval training (Wadley et al., 2015). Similarly, although MMP-9 and vWF have not been previously measured after HIIT, acute elevations in blood concentrations of vascular endothelial growth factor, hepatocyte growth factor, and endothelial microparticles have been observed (Wahl et al., 2014). Regarding the inflammatory response, a previous study has found that a single HIIT session was associated with immediate increases in blood concentrations of MCP-1 (Zwetsloot et al., 2014). However, a study by Kaspar et al. (2016) reported no change in blood concentrations of MCP-1 after HIIT. The reasons for the discrepancy between the current findings and those of Kaspar and colleagues are unclear but may be related to sample timing and study cohort; participants were predominantly female, and the authors sampled athletes at 30-min post HIIT. Conversely, our cohort comprised only males, and blood was sampled immediately post HIIT. Despite this, our findings add to the emerging body of evidence that supports a role for physical activity in the modulation of inflammation, vascular endothelial function, and oxidative metabolism (Bruunsgaard, 2005).

As opposed to the first training session, T-tau concentrations did not significantly change in response to acute exercise at session 6. In addition, after 2 weeks of training we observed significant attenuation in the acute post-exercise increases of NRGN, PRDX-6, MMP-9, and vWF. That we found no difference in the acute response of MCP-1 at session 1 vs. session 6 is in agreement with the findings of Zwetsloot et al. (2014) using a similar HIIT protocol, over the same time frame. Furthermore, while there have been no other assessments of PRDX-6, MMP-9, and vWF in response to multiple HIIT training bouts, a previous study found that a 4-week HIIT training protocol in elevated temperatures was associated with increased lipid peroxidation and decreased protein oxidation in response to an acute bout of maximal intensity exercise (Souza-Silva et al., 2016). In addition, a previous study using cardiac transplant patients found that 8 weeks of HIIT training significantly improved endothelial function and was associated with lower resting levels of pro-atrial natriuretic peptide and C-reactive protein (also an index of inflammation) (Hermann et al., 2011). However, given the lack of studies which have evaluated the acute biomarker-response to a single bout of HIIT after a period of training, and the scarcity of reports that have evaluated the biomarkers measured in the current study, interpretation is difficult. Still, our results suggest that a training period of 2 weeks is sufficient to augment the acute response to a single high-intensity exercise bout in numerous biomarkers related to brain injury.

The mechanism(s) underlying the exercise-induced changes in biomarker concentrations observed after HIIT in the current study remain unclear. While there is substantial literature on the effects of exercise on inflammation (Febbraio, 2007; Woods et al., 2009) and vascular endothelial activity (Inkabi et al., 2017), the source and mechanism(s) by which brain-derived proteins (NRGN, VILIP-1, s100B, NSE, PRDX-6, CKBB, and T-tau) appear in the blood after exercise are not well understood. Indeed, despite being predominantly expressed in brain tissue, each of the biomarkers measured in the current study have been identified in extracranial tissues (Tsung, 1976; Lubke et al., 1994; Muley et al., 2003; Laterza et al., 2006; Nielsen et al., 2009; Hampel et al., 2010; Buonora et al., 2015; Isgro et al., 2015; Kawata et al., 2016). For example, it has been suggested that increases in blood concentrations of s100B are derived from skeletal muscle in response to damage incurred through physical exertion (Stocchero et al., 2014; Riuzzi et al., 2017). However, this does not negate the possibility that these molecules may have been released from the central nervous system in response to physical activity; experimental studies have shown that exercise increases both glympathic influx and clearance (He et al., 2017; von Holstein-Rathlou et al., 2018). S100B has also been used as a metric of BBB permeability, which may also be influenced by exercise (Marchi et al., 2013; Koh and Lee, 2014). Insofar as these proposed mechanisms (glympathic clearance and BBB disruption) are also implicated in concussion, when concussive brain injury occurs in concert with physical exertion, the interpretation of biomarker findings will remain difficult. Yet, that exercise and mTBI may yield similar biomarker responses does not negate the utility of biomarkers in studying brain injury. For example, cardiac troponin (cTn) is an internationally recognized clinical diagnostic surrogate for acute myocardial infarction (MI) (Lindbloom and Stevermer, 1999; Vasikaran et al., 2012; Neumann et al., 2016; Pickering et al., 2016), yet recent studies have found that cTn, alongside other biomarkers associated with cardiovascular disease, can be elevated in the blood after exercise in the absence of a cardiac event or any apparent negative health occurrence (Saenz et al., 2006; Shave et al., 2010). Hence, future studies are needed to disentangle the biomarker profiles of exercise and mTBI, possibly through establishing concentration cutoffs that may separate exercise from injury, as has been previously proposed (Kiechle et al., 2014), or through focused investigations on the underlying mechanisms of both conditions.

The results of the current study should be interpreted within the context of its limitations. The sample size was limited, and our cohort was restricted to male participants. Hence, we were unable to address potential sex differences in the biomarker response to exercise. In addition, blood samples were taken only immediately post-exercise; more sample time-points would permit greater characterization of the temporal kinetics of for each biomarker and would allow for more accurate comparisons to previous studies that varied in sample acquisition time. Moreover, the addition of a control group matched for the habitual activities of the HIIT group would have aided in mediating any potential biological confounds outside of exercise that may impact blood biomarker concentrations. Despite this, we were able to discern robust changes in specific biomarker levels in response to an acute bout of HIIT and identified significant basal and exercise-induced differences in this response after a modest, 2-week training protocol.

Blood biomarkers commonly associated with brain injury are significantly elevated in response to a single bout of HIIT. This response is attenuated for some, but not all markers after a 2-week, 6-session high-intensity training protocol. While the results of this study do not negate the importance of blood biomarkers as a tool in concussion research, future studies investigating concussion in populations where injury commonly occurs during physical exertion cannot ignore this potential confound. Future research is necessary to further characterize the biological sequelae to both exercise and brain injury.

## Author Contributions

AD, SR, ST, and MS made substantial contributions to the conception or design of the work. AD, MS, KM, NC, and MH were involved in acquisition, analysis, and interpretation of data. AD, MH, KM, and SR drafted the work. All authors revised the manuscript for important intellectual content and approved the final version.

## Conflict of Interest Statement

The authors declare that the research was conducted in the absence of any commercial or financial relationships that could be construed as a potential conflict of interest.
