# Supplementary material for: High-Intensity Interval Training Is Associated With Alterations in Blood Biomarkers Related to Brain Injury
Source: Front Physiol. 2018 Sep 28;9:1367. doi: 10.3389/fphys.2018.01367 (PMC6172320; doi:10.3389/fphys.2018.01367)
Supplement: Supplementary file 1 [file Table_1.DOCX]

| **Biomarker** | **HIIT Session 1** | | **HIIT Session 6** | |
| --- | --- | --- | --- | --- |
|  | **Pre (n = 11)** | **Post (n = 11)** | **Pre (n = 11)** | **Post (n = 11)** |
| s100B | 11 (100) | 11 (100) | 11 (100) | 11 (100) |
| NSE | 11 (100) | 11 (100) | 11 (100) | 11 (100) |
| GFAP | 5 (45.4) | 7 (63.6) | 2 (18.2) | 3 (27.3) |
| T-Tau | 11 (100) | 11 (100) | 11 (100) | 11 (100) |
| CKBB | 10 (90.9) | 11 (100) | 11 (100) | 11 (100) |
| NRGN | 11 (100) | 11 (100) | 11 (100) | 11 (100) |
| VILIP-1 | 10 (90.9) | 10 (90.9) | 10 (90.9) | 10 (90.9) |
| BDNF | 11 (100) | 11 (100) | 11 (100) | 11 (100) |
| PRDX-6 | 11 (100) | 11 (100) | 11 (100) | 11 (100) |
| MCP-1 | 11 (100) | 11 (100) | 11 (100) | 11 (100) |
| MMP-9 | 11 (100) | 11 (100) | 11 (100) | 11 (100) |
| vWF | 11 (100) | 11 (100) | 11 (100) | 11 (100) |

**Supplementary Table 1**. Percent of Samples Detectable

Values presented as the number of samples and percent (%).

Detectable samples are within the assay limits of detection and display a coefficient of variation < 25% between sample replicates.

s100 calcium binding protein beta (s100B); neuron-specific enolase (NSE); peroxiredoxin (PRDX); glial fibrillary acidic protein (GFAP); brain derived neurotrophic factor (BDNF); matrix metalloproteinase (MMP); monocyte chemoattractant protein (MCP); total tau (T-Tau); creatine kinase-BB isoenzyme (CKBB); neurogranin (NRGN); visinin-like protein (VILIP); von Willebran factor (vWF).
