# Supplementary material for: High-Intensity Interval Training Is Associated With Alterations in Blood Biomarkers Related to Brain Injury
Source: Front Physiol. 2018 Sep 28;9:1367. doi: 10.3389/fphys.2018.01367 (PMC6172320; doi:10.3389/fphys.2018.01367)
Supplement: Supplementary file 2 [file Table_2.DOCX]

| **Marker** | **Intraassay %CV** | **Interassay %CV** |
| --- | --- | --- |
| S100B | 4.15 | 11.69 |
| GFAP | 8.31 | 15.10 |
| NSE | 8.69 | 14.67 |
| BDNF | 5.14 | 12.26 |
| NRGN | 6.99 | 12.76 |
| PRDX-6 | 3.43 | 14.90 |
| CKBB | 7.46 | 16.76 |
| VILIP-1 | 6.98 | 14.92 |
| vWF | 6.60 | 14.08 |
| MCP-1 | 7.00 | 11.75 |
| MMP-9 | 8.89 | 16.91 |
| T-tau | 6.25 | 10.68 |

**Supplementary Table 2.** Biomarker Assay Coefficients of Variation

**Abbreviations**: s100 calcium binding protein beta (S100B), glial fibrillary acidic protein (GFAP), neuron-specific enolase (NSE), brain-derived neurotrophic factor (BDNF), neurogranin (NRGN), peroxiredoxin (PRDX)-6, creatine kinase-BB isoenzyme (CKBB), visinin-like protein (VILIP)-1, von Willebrand factor (vWF), monocyte chemoattractant protein (MCP)-1, matrix metalloproteinase (MMP)-9, and total tau (T-tau).

**Calculation of intra- and inter-assay coefficients of variation (CV)**

To control for potential analytic error, intra- and inter-assay coefficients of variation (CVs) were calculated to quantify precision of biological measures. The CV is expressed as a percentage of deviation from the mean. Intra-assay %CV was calculated as the ratio of the pooled standard deviation from all samples (each analyzed in duplicate) and the overall mean, and then multiplied by 100. Inter-assay %CV refers to assay-to-assay consistency that was calculated using the pooled standard deviation divided by the overall mean of all duplicated samples, and then multiplied by 100. For all biomarkers assayed, values were included if replicates had a coefficient of variation (CV) of less than a 25% and had an inter-plate variance of less than 25% as measured by internal controls.
