# Supplementary material for: High-Intensity Interval Training Is Associated With Alterations in Blood Biomarkers Related to Brain Injury
Source: Front Physiol. 2018 Sep 28;9:1367. doi: 10.3389/fphys.2018.01367 (PMC6172320; doi:10.3389/fphys.2018.01367)
Supplement: Supplementary file 3 [file Table_3.DOCX]

| **Biomarker** | **Pre vs. Post**  **HIIT Session 1** | **Pre vs. Post**  **HIIT Session 6** | **HIIT Session 1 vs. 6**  **Δ Pre vs. Post** | **Session 1 vs. 6**  **Pre-HIIT** |
| --- | --- | --- | --- | --- |
| s100B | 4.7^***^ | 3.1^**^ | -2.2^*^ | -2.1^*^ |
| NSE | 11.3^***^ | 4.3^***^ | -0.6 | -3.0^**^ |
| T-tau | 6.7^***^ | 1.7 | -3.5^***^ | 1.6 |
| CKBB | 3.9^***^ | 4.0^***^ | -1.4 | -0.1 |
| NRGN | 9.2^***^ | 5.8^***^ | -5.2^***^ | -1.5 |
| VILIP-1 | 1.4 | 1.6 | -0.4 | 2.7^*^ |
| BDNF | 6.3^***^ | 2.8^*^ | -1.9 | -0.2 |
| PRDX-6 | 8.8^***^ | 3.6^***^ | -6.1^***^ | -0.8 |
| MCP-1 | 5.1^***^ | 4.8^***^ | -1.4 | -2.1^*^ |
| MMP-9 | 6.6^***^ | 4.9^***^ | -2.9^**^ | -2.4^*^ |
| vWF | 5.9^***^ | 5.4^***^ | -4.4^***^ | 0.7 |

**Supplementary Table 3.** Biomarker effect sizes

Biomarker effect sizes are calculated as the bootstrapped ratio: the mean of biomarker loadings

divided by the standard error of the mean. The direction of the effect size relates to its

covariance with the outcome; large positive effect sizes = positive relationship, large negative

effect size = large negative relationship.

Δ Pre vs. Post; the difference score calculated for each biomarker by subtracting the pre-HIIT value from the post-HIIT value.

s100 calcium binding protein beta (s100B); neuron-specific enolase (NSE); peroxiredoxin (PRDX); glial fibrillary acidic protein (GFAP); brain derived neurotrophic factor (BDNF); matrix metalloproteinase (MMP); monocyte chemoattractant protein (MCP); total tau (T-Tau); creatine kinase-BB isoenzyme (CKBB); neurogranin (NRGN); visinin-like protein (VILIP); von Willebran factor (vWF).

^*^ significant at p < 0.05

^**^ significant at p < 0.005

^***^ significant at p < 0.0005
