# Supplementary material for: High-Intensity Interval Training Is Associated With Alterations in Blood Biomarkers Related to Brain Injury
Source: Front Physiol. 2018 Sep 28;9:1367. doi: 10.3389/fphys.2018.01367 (PMC6172320; doi:10.3389/fphys.2018.01367)
Supplement: Supplementary file 4 [file Table_4.DOCX]

| **Biomarkers** | **Δ HIIT Pre-Post Session 1** | **Δ HIIT Pre-Post Session 6** |
| --- | --- | --- |
| s100B | 473.3 (210.9 - 1238.7) | 222.5 (76.5 - 525.4) |
| NSE | 695.0 (579.8 - 900.6) | 614.5 (211.0 - 963.5) |
| T-Tau | 5.5 (3.4 - 10.7) | 1.6 (-0.1 - 3.1) |
| CKBB | 565.1 (316.7 - 722.2) | 215.3 (200.5 - 311.9) |
| NRGN | 4866.2 (1890.8 - 8070.6) | 493.2 (311.4 - 2325.0) |
| VILIP-1 | 1.8 (-0.4 - 4.8) | 2.1 (0.2 - 3.4) |
| BDNF | 1066.5 (757.3 - 3154.1) | 305.9 (52.6 - 2513.7) |
| PRDX-6 (ng/mL) | 16.1 (11.5 - 33.5) | 4.5 (1.7 - 10.3) |
| MCP-1 | 30.3 (14.8 - 58.3) | 16.0 (10.5 - 51.1) |
| MMP-9 (ng/mL) | 29.5 (23.7 - 39.2) | 19.3 (6.2 - 22.3) |
| vWF (µg/mL) | 8.4 (5.8 - 13.6) | 4.0 (1.4 - 4.3) |

**Supplementary Table 4.** Pre-post changes in biomarker concentrations

Biomarker concentrations reported in pg/mL unless otherwise stated

Δ represents the change in biomarker concentrations pre vs. post exercise.

s100 calcium binding protein beta (s100B); neuron-specific enolase (NSE); peroxiredoxin (PRDX); glial fibrillary acidic protein (GFAP); brain derived neurotrophic factor (BDNF); matrix metalloproteinase (MMP); monocyte chemoattractant protein (MCP); total tau (T-Tau); creatine kinase-BB isoenzyme (CKBB); neurogranin (NRGN); visinin-like protein (VILIP); von Willebran factor (vWF).
